# Oculogyric Crisis and Suicidal Ideations in a Patient with Schizophrenia: A Case Report with Pharmacogenetic Findings

**DOI:** 10.1192/j.eurpsy.2025.2109

**Published:** 2025-08-26

**Authors:** D. Žujić, N. Božina, A. Mihaljević-Peleš, L. Ganoci, L. Šimićević, M. Živković

**Affiliations:** 1 School of Medicine, University of Zagreb; 2 Department of Psychiatry; 3 Department for Pharmacogenomics, University Hospital Centre Zagreb, Zagreb, Croatia

## Abstract

**Introduction:**

Suicidal ideations are severe and serious symptoms in the clinical presentation of schizophrenia, as well as adverse reactions such as oculogyric crises. In certain situations, they may be associated with specific pharmacogenetic factors, such as gene variations for the serotonin transporter (SERT) and the CYP2D6 enzyme.

**Objectives:**

A 37-year-old patient with treatment-resistant schizophrenia, characterized by frequent suicidal ideations, is regularly treated with clozapine (100 mg/day). Additionally, during disease exacerbations, the patient is given haloperidol (10 mg/day) as supplemental therapy, resulting in the development of oculogyric crises.

**Methods:**

Due to the lack of therapeutic response and a predisposition to side effects, a pharmacogenetic analysis revealed *CYP2D6* genotype *4/*9 and 5- *HTTLPR* genotype S_A_/S_A_. This indicates CYP2D6 intermediate enzyme activity and SERT low activity. Due to these findings, haloperidol was discontinued, and paliperidone palmitate was introduced at a dose of 75 mg monthly, after which the oculogyric crises no longer occurred. The pharmacogenetic results showed reduced SERT activity, which may be associated with the decreased therapeutic response to clozapine and the persistence of suicidal ideations.

**Results:**

Haloperidol is metabolized via CYP2D6, and its intermediate activity can lead to higher plasma concentration, resulting in extrapyramidal side effects such as oculogyric crises. Paliperidone is a metabolite of risperidone, and the activity of CYP450 enzymes has a minimal impact on its therapeutic response and potential for adverse reactions. HTTLPR regulates the transcriptional activity of the 5-HTT gene, so genotypes with low expressions, such as S’/S’ or S’/L’, may exhibit a weaker response to clozapine, which may include the persistence of suicidal ideations.

**Conclusions:**

The personalized antipsychotic treatment according to an individual’s pharmacogenetic profile may prevent adverse reactions and potentially explain therapeutic resistance in such cases where clozapine is otherwise indicated. Effective modern psychopharmacological treatment requires understanding pharmacogenetic factors and their influence on therapeutic response and the development of adverse reactions.

**Disclosure of Interest:**

None Declared

